# The Bone Extracellular Matrix in Bone Formation and Regeneration

**DOI:** 10.3389/fphar.2020.00757

**Published:** 2020-05-26

**Authors:** Xiao Lin, Suryaji Patil, Yong-Guang Gao, Airong Qian

**Affiliations:** Laboratory for Bone Metabolism, Xi'an Key Laboratory of Special Medicine and Health Engineering, Key Laboratory for Space Biosciences and Biotechnology, Research Center for Special Medicine and Health Systems Engineering, NPU-UAB Joint Laboratory for Bone Metabolism, School of Life Sciences, Northwestern Polytechnical University, Xi'an, China

**Keywords:** ECM, bone formation, bone tissue engineering, bone repair, bone cells

## Abstract

Bone regeneration repairs bone tissue lost due to trauma, fractures, and tumors, or absent due to congenital disorders. The extracellular matrix (ECM) is an intricate dynamic bio-environment with precisely regulated mechanical and biochemical properties. In bone, ECMs are involved in regulating cell adhesion, proliferation, and responses to growth factors, differentiation, and ultimately, the functional characteristics of the mature bone. Bone ECM can induce the production of new bone by osteoblast-lineage cells, such as MSCs, osteoblasts, and osteocytes and the absorption of bone by osteoclasts. With the rapid development of bone regenerative medicine, the osteoinductive, osteoconductive, and osteogenic potential of ECM-based scaffolds has attracted increasing attention. ECM-based scaffolds for bone tissue engineering can be divided into two types, that is, ECM-modified biomaterial scaffold and decellularized ECM scaffold. Tissue engineering strategies that utilize the functional ECM are superior at guiding the formation of specific tissues at the implantation site. In this review, we provide an overview of the function of various types of bone ECMs in bone tissue and their regulation roles in the behaviors of osteoblast-lineage cells and osteoclasts. We also summarize the application of bone ECM in bone repair and regeneration. A better understanding of the role of bone ECM in guiding cellular behavior and tissue function is essential for its future applications in bone repair and regenerative medicine.

## Introduction

Trauma, fractures, congenital disease, or tumors can cause bone defects that are challenging to heal. This is especially true for large bones, where the missing tissue is larger than the spontaneous healing ability of osteoblasts (El-Rashidy et al., 2017; Fabris et al., 2018). For small defects, autologous bone grafts remain the gold standard. This approach relies on bone tissue harvested from a patient's own donor site, which is transplanted into the same patient's damaged area. Because the grafts contain the native bone matrix, osteoblasts, and growth factors, they intrinsically possess osteoinductivity and osteoconductivity (Garcia-Gareta et al., 2015). However, this approach is limited by the available sources of grafts and secondary damage at the donor site. By contrast, while having similar biological characteristics and mechanical properties as autogenous bone, allogeneic bone carries the risk of transmission of infectious diseases and the possibility of immune rejection (Hinsenkamp et al., 2012).

In recent years, tissue engineering technology has enabled the production of artificial bone in large quantities. The resulting materials have the potential advantages of excellent biocompatibility, osteoinductivity, and osteoconductivity, providing a promising new method for bone repair. The manufacture of superior tissue-engineering constructs depends on three basic elements: appropriate scaffolds to support tissue-cell regeneration, cytokines, and appropriate seed cells. As the physical basis of artificial grafts, scaffold materials play a key role in the construction of artificial bone (Noori et al., 2017). Ideally, the scaffold material should mimic the characteristics of natural bone, providing a suitable biochemical environment and biomechanical support for the adhesion, migration, proliferation, osteogenic differentiation, and angiogenesis of seed cells on the scaffold. Finally, it must allow the gradual integration into the host tissue during the healing process, allowing it to bear normal loads (Mishra et al., 2016; Roseti et al., 2017). During bone regeneration, the homing of mesenchymal stem cells (MSCs), the formation of osteoblasts, extracellular matrix (ECM) and osteoid mineralization, and the formation of terminally differentiated osteocytes play an important role in bone formation (Wang et al., 2013).

The ECM is a non-cellular three-dimensional structure secreted by cells into the extracellular space. It is composed of specific proteins and polysaccharides. The ECM of each tissue type has a unique composition and topology during development (Frantz et al., 2010). The ECM provides the tissue with integrity and elasticity, and it is constantly being reformed due to changes in the abundance of receptors, growth factors, and the pH of the local environment to control the development, function, and homeostasis of tissues and organ (Bonnans et al., 2014; Mouw et al., 2014). The ECM is considered to represent the fourth element in the development of bone tissue engineering (Ravindran et al., 2012). The bone matrix comprises organic (40%) and inorganic compounds (60%). Moreover, its exact composition differs based on sex, age, and health conditions. The main inorganic components of the ECM are calcium-deficient apatite and trace elements. By contrast, the organic ECM is significantly more complex consists mainly of collagen type I (90%), and noncollagenous proteins (10%). It is mainly synthesized by osteoblasts before the mineralization process (Mansour et al., 2017). The non-collagenous proteins can be classified into four groups: γ-carboxyglutamate-containing proteins, proteoglycans, glycoproteins, and small integrin-binding ligands N-linked glycoproteins (SIBLINs) (Paiva and Granjeiro, 2017). Bone ECM dynamically interacts with osteoblast-lineage cells and osteoclasts to regulate the formation of new bone during regeneration.

In this review, we briefly introduce the inorganic and organic ECM of bone tissue (**Table 1**), including collagenous and non-collagenous proteins, and summarize the effects of the ECM on osteoblast-lineage cells, including MSCs, osteoblasts, and osteocytes, and osteoclasts. Finally, the application of ECM-based scaffold for bone regeneration in bone tissue engineering is reviewed.

**Table 1:** The list of bone ECM components and their role in bone formation.

Bone ECM	Expressed from	Function in bone tissue	Reference
**Organic ECM**			
**Collagenous protein**			
*Type I collagen*	Osteoblast	–Scaffold for bone cells –Maintain bone strength	(Saito and Marumo, 2015)
		–Promote bone formation	(Fonseca et al., 2014)
**		–Regulate collagen fibrillogenesis	
Types III and V collagen	Bone	–Promote bone	(Garnero, 2015)
**Noncollagenous protein**			
**Proteoglycans**			
*Biglycan*	Osteoblast	–Promote collagen fibrillogenesis –Promote bone formation	(Moorehead et al., 2019)
*Decorin*	Osteoblast	–Promote collagen fibrillogenesis –Promote bone formation	(Coulson-Thomas et al., 2015)
*Keratocan*	Osteoblast	–Promote mineral deposition rates	
*Asporin*	Articular cartilage or periodontal tissue	–Promote collagen mineralization	(Kalamajski et al., 2009)
**γ-carboxyglutamic acid-containing proteins**			
*Osteocalcin*	Osteoblast	–Regulate calcium metabolism –Indicate bone formation	(Mizokami et al., 2017)
*Matrix* Gla Protein *(MGP*)	Osteoblast, osteocyte, and chondrocyte	–Inhibit bone formation and mineralization	(Kaipatur et al., 2008)
*Periostin*	Osteoblast and precursor cells	–Regulate collagen fibrillogenesis -Maintain bone strength	(Wen et al., 2018)
**Glycoproteins**			
*Osteonectin*	Osteoblast	-Promote bone formation and mineralization -Regulate collagen fibrillogenesis	(Rosset and Bradshaw, 2016)
		-Maintain biomechanical properties	(Delany et al., 2000)
*Thrombospondins*	Osteoblast	-Promote bone formation -Regulate collagen fibrillogenesis	(Delany and Hankenson, 2009)
*R-spondins*	Bone	-Promoter Wnt/β-catenin signaling -Regulate bone development	(Shi et al., 2017)
**Small integrin-binding ligand *N*-linked glycoproteins/SIBLINGs**			
*BSP*	Mineralized tissues	–Promote bone formation and mineralization	(Marinovich et al., 2016)
*OPN*	Osteoblast, odontoblast and osteocyte	–Promote bone formation and mineralization –Regulate bone remodeling	(Singh et al., 2018)
*DMP1*	Osteocyte and dentin	–Regulate phosphate metabolism –Promote bone mineralization	(Jani et al., 2016)
*MEPE*	Osteocyte and dentin	–Regulate phosphate metabolism	(Zelenchuk et al., 2015)
		–Promote bone mineralization	
**Inorganic ECM**			
*Hydroxyapatite*	Bone	–Biomineralization	(Tavafoghi and Cerruti, 2016)

## Major Components of Bone ECM

### Organic ECM

#### Collagenous Proteins

The collagen type I, III, and V are the most abundant constituents of the organic ECM in bones. The main function of collagens is mechanical support and to act as a scaffold for bone cells (Saito and Marumo, 2015). Type I collagen accounts for 90% of the total collagen in bone tissue and forms triple helices of polypeptides which form the collagen fibrils. These fibrils interact with other collagenous and noncollagenous proteins to assemble the higher-order fibril bundles and fibers (Varma et al., 2016). Collagen types III and V regulate the fiber diameter and fibrillogenesis of type I collagen and are present in smaller amounts (Garnero, 2015). The inter- and intra-chain crosslinks of collagen are key to its mechanical properties, which maintain the polypeptide chains in a tightly organized fibril structure. Collagen plays an important role in determining bone strength. The lack of type I collagen or mutation of collagen structure results in changes in the ECM, and thus significantly increases fracture risk (Fonseca et al., 2014).

#### Noncollagenous Proteins

##### Proteoglycans

Proteoglycans are characterized by the presence of glycosaminoglycan (GAG) residues covalently bound to the protein core. The six types of GAG residues found in proteoglycans include keratan sulfate, chondroitin sulfate, heparan sulfate, hyaluronic acid, and dermatan sulfate (Kjellen and Lindahl, 1991). Small leucine-rich proteoglycans (SLRPs), such as biglycan, decorin, keratocan, and asporin, are important proteoglycans family in the bone. SLRPs are secreted extracellular proteins that interact with cell surface receptors and cytokines to regulate both normal and pathological cellular behaviors. During bone formation, SLRPs participate in all stages including cell proliferation, osteogenesis, mineral deposition, and bone remodeling (Kirby and Young, 2018). In addition, SLRPs regulate the process of collagen fibrillogenesis, the dysregulation of which leads to defects in the organization and production of collagen, culminating in fibrosis due to either orthopedic injuries or genetic deficiencies (Moorehead et al., 2019). Biglycan and decorin are class I SLRPs that contain either dermatan or chondroitin sulfate GAG chains. Biglycan is expressed during the process of cell proliferation and mineralization, while Decorin is continuously expressed starting from bone matrix deposition. Keratocan is mainly expressed in osteoblasts and involved in regulating bone formation and mineral deposition rates (Coulson-Thomas et al., 2015). Asporin, another member of SLRP, has been shown to bind with type I collagen to promote collagen mineralization (Kalamajski et al., 2009). Therefore, SLRPs play an essential role to maintain bone homeostasis.

##### γ-Carboxyglutamic Acid-Containing Proteins

One important group of bone ECM proteins contains γ-carboxyglutamic acid (Gla), a specific modified glutamic acid produced by a vitamin K-dependent post-translational modification. These proteins are mainly present in the serum, bone matrix, dentin, and other calcified tissues (Finkelman and Butler, 1985). The main Gla-containing proteins in the bone are osteocalcin (OCN), matrix Gla protein (MGP), and periostin (Wen et al., 2018). OCN is specifically expressed by bone-forming osteoblasts and contains three Gla residues, which give OCN the ability to bind calcium to modulate calcium metabolism by mediating its association with hydroxyapatite. The bone resorption process reduces OCN's affinity for hydroxyapatite, thereby enhance the release of OCN into circulation. Circulating OCN not only acts as a hormone that regulates glucose and energy metabolism, but its concentration in serum can be used as a biochemical indicator of bone formation (Mizokami et al., 2017). MGP is a 14-kDa extracellular protein that synthesized by osteoblasts, osteocytes, and chondrocytes in the bone. MGP-deficient mice have reportedly exhibited premature bone mineralization, while mice with MGP overexpression in osteoblasts showed reduced mineralization of intramembranous bone and hypomineralized tooth dentin and cementum (Luo et al., 1997; Kaipatur et al., 2008). Obviously, MGP is responsible for disrupting bone formation and inhibiting mineralization.

Except for OCN and MGP, periostin is another abundantly expressed Gla-containing protein in bone. Periostin is mainly secreted by osteoblasts and their precursor cells in long bones and is also found in other organs, such as the heart (Wen et al., 2018). Structurally, periostin comprises four domains, a signal sequence, a cysteine-rich emilin-like (EMI) domain, four repetitive and conserved FAS-1 domains, and a variable hydrophilic C-terminal domain, each of which provides different functions, such as FAS-1 providing cell adhesion ability (Merle and Garnero, 2012). As an adhesion molecule, periostin promotes aggregation, adhesion, proliferation, and differentiation of osteoblasts by binding to cell surface receptors. Moreover, periostin participates in collagen folding and fibrillogenesis, which is essential for matrix assembly and further maintains bone strength (Wen et al., 2018).

##### Glycoproteins

Glycoproteins contain covalently attached carbohydrate molecules on the protein chain in various combinations and positions. Of glycoprotein in the bone matrix, osteonectin, also known as secreted protein acidic and rich in cysteine (SPARC), is a common representative. It is present in mineralized tissues and highly expressed in osteoblasts of bone. Osteonectin is a vital regulator of the calcium release by binding collagen and HA crystals, thereby influencing the mineralization of collagen during bone formation (Rosset and Bradshaw, 2016). With experiments *in vivo*, Delany et al. (2000) demonstrates that osteonectin-null mice had lower total collagen I content, bone mineral density and numbers of osteoblasts and osteoclasts in bone, and exhibited reduced biomechanical properties. Thus, osteonectin takes part in regulating bone remodeling and maintaining bone mass. Thrombospondins (TSPs), which are classified as TSP1 through TSP5, are present in developing skeleton and bone and is expressed by osteoblasts. In mice, global knockout of TSP-1, -3, and -5 can cause severe abnormalities in skeletal development (Delany and Hankenson, 2009). Moreover, TSP1-null mice show the increased bone mass and cortical bone size, and the differentiation of osteoblast is promoted, which is partly by activating latent TGF-β (Amend et al., 2015). TSP2-null mice have enhanced cortical bone density and osteoprogenitor numbers, combined with the abnormality of collagen fibrillogenesis (Hankenson et al., 2000). These indicate that TSPs play a critical role in bone cell differentiation and maintaining bone mass. R-spondins (roof plate-specific spondin) are a group of four secreted homologous glycoproteins (Rspo1-4) that belong to thrombospondin repeat containing matricellular protein family. They are widely expressed at different stages of skeletal tissue and act as a reinforcer of the Wnt/β-catenin signaling pathway through leucine-rich repeat-containing G-protein-coupled receptors 4, 5, and 6 (Lgr4/5/6). In bone tissue, R-spondins are identified as regulators of the skeleton that control embryonic bone development and adult bone remodeling (Shi et al., 2017).

##### Small Integrin-Binding Ligand N-Linked Glycoproteins/SIBLINGs

SIBLINGs are a family of glycophosphoproteins that includes bone sialoprotein (BSP), osteopontin (OPN), dentin matrix protein-1 (DMP1), dentin sialophosphoprotein (DSPP), and matrix extracellular phosphoglycoprotein (MEPE). These proteins are predominantly found in mature, mineralized tissues, such as dentin and bone (Bellahcene et al., 2008).

BSP is a highly glycosylated noncollagenous phosphoprotein, that is expressed at the beginning of hard connective tissue mineralization. As a result of the deletion of BSP in mice, cementum deposition is significantly reduced, and long bone length and cortical thickness, the rate of bone formation are also reduced. Thus, BSP is vital in the regulation of osteoblast differentiation and initiation of matrix mineralization in bone tissue (Marinovich et al., 2016). Like BSP, OPN is a major regulator of bone formation, mineralization, especially in bone turnover. It is highly expressed by osteoblasts, odontoblasts, and osteocytes. OPN is abundant in serine-, acidic, and aspartate-rich motif, which are potential phosphorylation sites involved in inhibiting mineralization. In bone remodeling, OPN regulates osteoclastogenesis and osteoclast activity, which contributes to bone formation and resorption (Singh et al., 2018).

DMP1 and MEPE are mainly produced by fully differentiated osteoblasts in bone, and also expressed by pulp cells and odontoblasts. DSPP is important for the mineralization of tooth dentin, and is consequently abundant in dentin tissue (Bouleftour et al., 2019). Mice lacking DMP1 show severe bone defects, displaying increased serum fibroblast growth factor 23 (FGF23) and decreased serum phosphorus, as well as deformed and low-mineralized bone (Jani et al., 2016). Knockout of MEPE in mice increases bone mass and trabecular density and shows abnormal cancellous bone. Moreover, MEPE interacts with DMP1 and PHEX to affect FGF23 expression, thereby regulating phosphate, mineralization, and bone turnover (Zelenchuk et al., 2015). DMP1 and MEPE, thus, appear as key regulators of matrix mineralization and phosphate metabolism.

### Inorganic ECM

The main inorganic constituent of hard tissues, such as bone and dentine, is hydroxyapatite (HA, Ca_5_(PO_4_)_3_OH) (Ramesh et al., 2018). The deposition of HA occurs through the process called biomineralization. Interactions between minerals and matrix in teeth and bones, such as amino acids present in non-collagenous proteins, control HA formation. Collagen is produced during the mineralization of tissue and acts as a template for the deposition of HA (Tavafoghi and Cerruti, 2016). Due to the significant chemical and physical resemblance of HA to the mineral constituents of human bones and teeth, it is both biocompatible and osteoconductive. Consequently, HA is widely used for coatings on metallic implants, bone fillings, and injectable bone substitutes (Ramesh et al., 2018).

## Function of the Bone ECM in Osteoblast-Lineage Biology

Osteoblast-lineage cells are bone-forming cells in bone remodeling. Osteoblasts develop from multipotent mesenchymal stem cells (MSCs), which can be isolated from the bone marrow or other tissues. The osteogenic differentiation of MSCs can be divided into four steps: (i) the commitment step produces lineage-specific progenitor cells; (ii) the proliferative phase of osteoprogenitors, in which genes associated with the cell cycle and histone signals are expressed; (iii) the phase of ECM secretion and morphological changes of immature osteoblasts; (iv) osteoid mineralization initiated by mature osteoblasts, which become terminally differentiated osteocytes (Paiva and Granjeiro, 2017). MSCs, osteoblasts, and osteocytes sense mechanical and biochemical signals from the ECM and respond to these signals by regulating their fate (Assis-Ribas et al., 2018).

### Regulation of BMSCs by the ECM

BMSCs are capable of migration, proliferation, differentiation, and cell-cell communication. Moreover, they can synthesize copious amounts of extracellular matrix proteins such as collagen type IIIα1 and Vα1, α5 and β5 integrin chains, fibronectin, connective tissue growth factor, and transforming growth factor beta I (TGFβI) (Ren et al., 2011). These are considered to be important for MSC homing and fate determination, such as adhesion, expansion, and spreading, through integrin receptors.

As an osteoblastic agent, TGFβ is coupled to the bone ECMs and moderately regulates the differentiation of early BMSCs into matrix-producing osteoblasts and osteocyte. Biglycan is can regulate the biological activity of TGF-β as well as matrix organization by binding to collagen type I. It has been reported that BMSCs isolated from biglycan-KO mice produced low amounts of collagen type I and showed a reduced response to TGF-β. Moreover, the deficiency of biglycan disrupts the ability to produce BMSCs, and also attenuates it's normal metabolic activity. In addition, biglycan-KO mice show the low activity of alkaline phosphatase (ALP)-positive MSCs, possibly due to apoptosis, which leads to a decrease of proliferation (Chen et al., 2002). In mice lacking biglycan and decorin (another member of the SLRP family), high concentrations of TGF-β activate downstream signaling pathways that stop the proliferation and induce the apoptosis of BMSCs. Therefore, decorin and biglycan mediate the proper sequestration of TGF-β and play a vital role in regulating the survival and growth of BMSCs (Bi et al., 2005).

Besides proteoglycans, glycoprotein TSP1 is also a major regulator of TGF-β activation and critical for regulation of the behaviors of MSCs inside the adult bone marrow niche microenvironment. In MSCs, TSP1 inhibits MSCs osteogenesis with decreased expression of Runx2 and ALP expression. This inhibition is due to latent TGF-β activation in MSCs, since anti-TGF-β antibody increased ALP activity in the presence of TSP1 (Bailey Dubose et al., 2012). Furthermore, the TSP1 effect on MSC proliferation has been reported to be mediated by activation of endogenous TGFβ in a dose-dependent manner. By contrast, the proliferation of MSC is not affected by TSP2, which can't activate TGFβ (Belotti et al., 2016). Therefore, TGFβ acts as an intermediary of TSP1 activity on MSCs.

Type I collagen fibrils in bone ECM also modulate osteogenesis by binding with integrins of osteoblast progenitors, which leads to initiated osteoblast differentiation cascade through Runx2 transcriptional activation (Elango et al., 2019). Fibrillogenesis starts from the interaction between type I and type V collagen, and then forms linear fibril. SLRP and thrombospondins can regulate collagen assembly by interacting with collagen fibrils. In mice, deletion of TSP2 results in increased number and proliferation ability of MSC, and also characterized by delayed osteogenesis and increased adipogenesis (Hankenson et al., 2000). Deficiency of TSP2 inhibits the differentiation of primary MSCs into osteoblasts, accompanied by decreased matrix collagen content and disrupted type I collagen assemble process (Alford et al., 2013). These results suggest that, unlike TSP1, TSP2 may act as an inhibitor of MSCs proliferation and a promoter of differentiation by regulating the mechanism of collagen fibrillogenesis.

Other ECM molecules, such as OPN, OCN, and DMP1, can regulate the proliferation of MSCs and osteogenesis. OPN increases the proliferation capacity of MSCs in a dose-dependent manner. On the other hand, OCN promotes the differentiation of MSCs into osteoblasts, with the increase of extracellular calcium levels, ALP activity, and the mRNA expression of OPN and OCN (Carvalho et al., 2019a). Numerous studies find that cytoskeleton and chromatin organization can affect cell migration. Liu and colleagues indicate that F-actin cytoskeleton and chromatin structure organized by EZH2-mediated H3K27me3 involves OPN-induced MSCs migration (Liu et al., 2018; Liu et al., 2019). In addition to stimulating the maturation of osteoblasts and osteocytes, DMP1 can also affect the pluripotency of MSCs. When DMP1 is removed, MSCs increasingly differentiate into osteogenic cells and bone mass, suggesting that it is a negative regulator of MSC differentiation (Zhang S. F. et al., 2018). Taken together, ECM that participates in bone formation and mineralization also significantly contributes to the growth, survival, and differentiation of MSCs (**Table 2**).

**Table 2:** Function of the bone ECM in MSCs.

Bone ECM	Functions in MSCs	Mechanism	Cell/Mice model	Reference
Biglycan	BMSCs production and proliferation (+)	Regulate amounts of collagen type I and response to TGF-β	Biglycan^−/−^ mice	(Chen et al., 2002)
Biglycan and Decorin	BMSCs survival and growth (+)	Regulate response to TGF-β	Biglycan Decorin DKO mice	(Bi et al., 2005)
TSP1	MSC osteogenesis (−); MSC proliferation (+)	Latent TGF-β activation	MSCs	(Bailey Dubose et al., 2012; Belotti et al., 2016)
TSP2	MSC number and proliferation ability (−); MSC osteogenesis (+)	Regulate collagen fibrillogenesis	TSP2^−/−^ mice	(Hankenson et al., 2000; Alford et al., 2013)
OPN	MSC proliferation capacity (+); MSCs migration (+)	Regulate F-actin cytoskeleton and chromatin structure	MSCs	(Carvalho et al., 2019a; Liu et al., 2019)
OCN	MSC osteogenesis (+)	Increase extracellular calcium and ALP	MSCs	(Carvalho et al., 2019a)
DMP1	MSCs pluripotency (+); MSC osteogenesis (−)	–	*Prx1-cre*; *DMP1^fl/fl^* mice	(Zhang S.F. et al., 2018)

DKO, double knockout.

### Regulation of Osteoblasts by the ECM

Immature and mature osteoblasts are the intermediate cells during MSCs osteogenesis. It continues the process of differentiation, along with the secretion of ECM and osteoid mineralization. Osteoblasts require a surface to synthesize new matrix, which is provided by collagen. If there is no substrate, osteoblasts synthesize a matrix that is only organized in the short range. Thus, this organized surface is used by osteoblasts to deposit mechanically stable and correctly structured bone tissue (Kerschnitzki et al., 2011). Different structures composed of type I collagen have different effects on the behavior of osteoblasts. In contrast to soluble and fibrillar forms, denatured forms of type I collagen inhibit the proliferation of osteoblast-like cells and can stimulate osteoblastic differentiation (Tsai et al., 2010). A small amount of type III collagen is also found in collagen fibrils of bone. Type III collagen null mice show affected osteoblast differentiation, consistent with decreased ALP activity, reduced osteogenic markers (OCN and BSP), and mineralization capacity (Volk et al., 2014). Therefore, collagen acts as a tissue scaffold, providing a matrix for anchoring cells and regulating the growth and osteogenic properties of osteoblasts.

Part of ECM protein not only regulates collagen fibrillogenesis but is required for osteoblast lineage progression, which ultimately affects mineralization. The contributions of osteonectin, keratocan, TSP1, and TSP2 to collagen fibrillogenesis have been extensively reported. In terms of influencing the maturation and function of osteoblasts, osteonectin and keratocan-null mice show fewer osteoblasts and decreased mineralized nodules in mutant cells (Igwe et al., 2011; Rosset and Bradshaw, 2016). TSP1 inhibits the mineralization of osteoblast *in vitro* and *in vivo* (Ueno et al., 2006). However, TSP2 promotes osteoblast mineralization by promoting the organization of osteoblast-derived ECM (Alford et al., 2010). Collectively, those proteins mediate the mineralization of osteoblasts through regulating collagen fibrillogenesis to some extent.

ECM molecules BSP and OPN are two SIBLINGs that contribute to the regulation of osteoblasts. BSP is crucial for the synthesis of the ECM and HA nucleation activity. It can promote osteoblast differentiation and enhance early bone mineralization to produce new bone *in vivo*. Especially the RGD sequence of BSP, which mediates the osteoblast behaviors by FAK and other extracellular kinases (Holm et al., 2015). By contrast, OPN can inhibit the process of osteoblast osteogenesis through inhibition of BMP-2, and act as a mineralization inhibitor of osteoblast in a phosphate-dependent manner (Huang et al., 2004; Singh et al., 2018). Consistent with that of OPN, OCN, which is produced by osteoblast, is considered as an inhibitor of bone mineralization. Osteocalcin null mice show larger HA crystal size, suggesting that osteocalcin may regulate the maturation rate of minerals (Zoch et al., 2016).

The Wnt pathway is an important regulatory for bone formation. Three ECM molecules, MGP, R-spondin2, and periostin, have been identified to modulate the mineralization of osteoblast through Wnt signaling. Knockdown of MGP inhibits the differentiation and mineralization of osteoblasts *via* up-regulating Wnt/β-catenin signaling pathway. Consistent with the results of *in vivo* experiment that overexpression of MGP inhibits the decreased bone mineral density induced by ovariectomy (Zhang J. et al., 2019). As a wnt agonist, R-spondin2 is abundantly expressed in pre-osteoblasts stimulated by Wnt. R-spondin2 promotes osteoblastogenesis *in vitro* and bone mass *in vivo*, supporting its vital role in osteoblastogenesis and bone development (Knight et al., 2018). Sclerostin is an important inhibitor of WNT/β-catenin signaling and regulates osteoblast matrix generation. It has been reported that periostin may interact directly with sclerostin and promotes Wnt signaling inhibited by sclerostin (Bonnet et al., 2016). Moreover, periostin can also affect osteoblast differentiation and bone formation, suggesting that periostin is involved in bone anabolism by regulating Wnt/β‐catenin signaling (Merle and Garnero, 2012) (**Table 3**).

**Table 3:** Function of the bone ECM in osteoblasts.

Bone ECM	Functions in osteoblasts	Mechanism	Cell/Mice model	Reference
Type I collagen	Osteoblast proliferation (−) Osteogenesis (+)	Denatured forms of collagen	MG63 cells	(Tsai et al., 2010)
Type III collagen	Osteogenesis and mineralization (+)	Regulate type I collagen, BSP, and OCN	Col3^−/−^ mice	(Volk et al., 2014)
Osteonectin	Osteoblast number and differentiation (+); bone formation (+)	Regulate collagen fibrillogenesis	Osteonectin^−/−^ mice	(Rosset and Bradshaw, 2016)
Keratocan	Osteoblast number and differentiation (+); bone formation (+)	Regulate collagen fibrillogenesis	Keratocan^−/−^ mice	(Igwe et al., 2011)
TSP1	Osteoblast mineralization (−)	Regulate collagen fibrillogenesis	MC3T3-E1 cells	(Ueno et al., 2006)
TSP2	Osteoblast mineralization (+)	Organization of osteoblast-derived ECM	MC3T3-E1 cells	(Alford et al., 2010)
BSP	Osteoblast differentiation and early bone mineralization (+)	FAK and other extracellular kinases	BSP^−/−^ mice	(Holm et al., 2015)
OPN	Osteoblast osteogenesis and mineralization (−)	BMP-2, phosphate-dependent manner	MC3T3-E1 cells	(Huang et al., 2004; Singh et al., 2018)
OCN	Osteoblast mineralization (−)	–	OCN^−/−^ mice	(Zoch et al., 2016)
MGP	Osteoblast differentiation and mineralization (+)	Wnt/β-catenin signaling pathway	MG63 cells	(Zhang J. et al., 2019)
R-spondin2	Osteoblast differentiation (+)	Wnt/β-catenin signaling pathway	*Ocn-Cre*; *Rspo2^fl/fl^* mice	(Knight et al., 2018)
Periostin	Osteoblast differentiation and bone formation (+)	Wnt/β-catenin signaling pathway		(Merle and Garnero, 2012)

### Regulation of Osteocytes by the ECM

Osteocytes are the terminally differentiated immobilized cells in the bone matrix. Although embedded in the bone matrix, osteocytes form contacts with each other and with bone lining cells, which aid bone growth and repair.

The bone matrix present around the intricate lacuno-canalicular network of osteocytes is continuously being resorbed and deposited in a process called perilacunar/canalicular remodeling (Dole et al., 2017). Changes in the overall formation rate of the canalicular network increase osteoblast activity and bone formation. Recently, it is demonstrated that the process by which osteocytes push type I collagen fibers outward from the center of the formed lacuna mediates osteocytes lacunae formation, which is accompanied by increased collagen deposition and collagen-fiber network compaction surround the lacunae. Therefore, the dynamic assembly of bone collagen contributes greatly to the encapsulation and mineralization of osteocytes in bone matrix (Shiflett et al., 2019).

Osteocytes can sense and respond to external mechanical cues. The stiffness of the surrounding matrix is one of the most important signals that regulate osteocyte behaviors, and changes in the stiffness of the ECM induce alterations in the cytoskeleton and cell morphology, as well as fibronectin, which leads to changes in paxillin and in turn affects the elongation of osteocyte gap junctions (Zhang D. M. et al., 2018). As osteocytes begin to expand processes and start mineralizing the neighboring matrix, the expression of DMP1 and MEPE is upregulated. The stiffness of the ECM, and especially that of the collagen-based substrates, affects DMP1 expression. The levels of DMP1 and Sclerostin are greatly increased on collagen-based substrates with low stiffness, indicating enhanced osteocyte differentiation compared to ECM substrates with high stiffness (Mullen et al., 2013). Changes of DMP1 levels mediate the sensing of mechanical stimuli by osteocytes, which may increase the attachment of osteocytes and remodeling of the matrix present inside the local microenvironment (Gluhak-Heinrich et al., 2003). In addition, DMP1 also inhibits the apoptosis of osteocytes, enhances bone mineralization, and prevents the disintegration of the osteocyte network (Dussold et al., 2019). MEPE is synchronized with DMP1 and differentially regulates bone remodeling by mechanical loading. MEPE knockout mice show increased bone mass, accompanied by suppressed mineralization, suggesting that both DMP1 and MEPE can regulate the mineralization in osteocytes and lacunar wall (Gluhak-Heinrich et al., 2007) (**Table 4**).

**Table 4:** Function of the bone ECM in osteocytes.

Bone ECM	Functions in osteocytes	Mechanism	Cell/Mice model	Reference
Type I collagen	Osteocyte mineralization (+)	Collagen deposition and collagen-fiber network compaction	GFP-col^+/−^/Dmp1-Cre^+/−^/tdTomato^+/−^ mice	(Shiflett et al., 2019)
DMP1	Osteocyte attachment (+); Osteocyte apoptosis (−)	External mechanical force	Col4a3^−/−^ mice	(Gluhak-Heinrich et al., 2003; Dussold et al., 2019)
MEPE	Osteocyte mineralization (+)	External mechanical force	MEPE^−/−^ mice	(Gluhak-Heinrich et al., 2007)

## Function of the Bone ECM in Osteoclasts

Osteoclasts, are multinucleated cells formed from the fusion and differentiation of monocyte/macrophage precursors, involve in bone resorption. The formation and activity of osteoclasts activated by macrophage colony-stimulating factor (M-CSF) and receptor for activation of nuclear factor κB (NF-κB) ligand (RANKL), which are derived from osteoblasts (Lin et al., 2019).

Upon osteoclast formation, TSP1, TSP2, MGP, and biglycan regulate osteoclast differentiation and resorption activity in different regulatory mechanisms. Both TSP1 and TSP2 are key positive regulators in osteoclast differentiation. TSP1 functions in the early stage of osteoclastogenesis, and TSP1 deficiency mice show decreased differentiation and activity of osteoclast. This is caused by increased inducible nitric oxide synthase (iNOS) (Amend et al., 2015). However, TSP2 induces osteoclastogenesis through NFATc1, which is a RANKL-dependent pathway, accompanied by an increased RANKL/OPG ratio (Wang et al., 2019). In contrast, MGP suppresses the nuclear translocation of NFATc1 and intracellular Ca^2+^ flux in osteoclasts, which in turns attenuate the differentiation and bone resorption. MGP also inhibits bone formation and MGP-null mice exhibit an osteopenic phenotype, suggesting that MGP plays a stronger role in bone absorption than in bone formation (Zhang Y. et al., 2019). With the same regulation mechanism as MGP, type I collagen can also act as an inhibitor of bone development by osteoclasts. The formation of osteoclasts can be suppressed by full length or 30–75 kDa fragments of type I collagen, which binds with the collagen receptor LAIR-1 and thereby maintaining bone strength (Boraschi-Diaz et al., 2018). TNFα has been shown to regulate osteoclast differentiation and survival in a RANKL-independent manner. In biglycan and fibromodulin double knockout mice, osteoclasts possess higher differentiation potential and surround with increased TNFα and RANKL cytokine. Exogenous biglycan or fibromodulin weakens the ability of osteoclast precursors to form TRAP-positive multinucleated cells. Therefore, biglycan alone or coupled with fibromodulin regulates osteclastogenesis through TNFα and/or RANKL to control bone mass (Kram et al., 2017).

The RGD sequence of OPN and BSP interact with αvβ3 integrin initiate osteoclast adhesion to bone matrix and formation of actin ring of polarized osteoclasts, which is crucial for bone development. Integrin-matrix combination is vital for podosome formation on osteoclasts. Thus, OPN plays a major role in osteoclast activity and sealing zone formation of osteoclasts (Singh et al., 2018). Moreover, OPN can be secreted by human osteoclasts in addition to osteoblast during bone resorption, which can be used as a chemokine for subsequent bone formation and resorption (Luukkonen et al., 2019). In addition, osteoclast surfaces and the number of osteoclasts are decreased in BSP knockout mice. BSP can promote bone resorption, and the migration of preosteoclast and mature osteoclasts is impaired in the absence of BSP (Boudiffa et al., 2010). OPN and BSP can act as a network to coordinate the function of osteoclasts. Osteoclasts derived from OPN and BSP double knockout mice exhibit higher number and resorption activity. The interaction between OPN/BSP and αVβ3 integrin may participate in determining osteoclast adhesion to bone matrix surface and subsequent resorption (Bouleftour et al., 2019) (**Table 5**).

**Table 5:** Function of the bone ECM in osteoclasts.

Bone ECM	Functions in osteoclasts	Mechanism	Cell/Mice model	Reference
TSP1	Osteoclast differentiation and activity (+)	Decrease inducible nitric oxide synthase (iNOS)	TSP1^−/−^ mice	(Amend et al., 2015)
TSP2	Osteoclastogenesis (+)	Transactivation of NFATc1; Increase RANKL/OPG ratio	RAW 264.7 cells	(Wang et al., 2019)
MGP	Osteoclast differentiation and bone resorption (−)	Suppress the nuclear translocation of NFATc1 and intracellular Ca^2+^ flux	MGP^−/−^ mice	(Zhang Y. et al., 2019)
Type I collagen	Osteoclast formation (−)	Bind with the collagen receptor LAIR-1	Primary BMMs	(Boraschi-Diaz et al., 2018)
Biglycan	Osteoclast precursors differentiation (−)	Decrease TNFα and RANKL cytokine	Biglycan Fibromodulin DKO mice	(Kram et al., 2017)
OPN	Osteoclast activity and sealing zone formation (+)	RGD sequence interact with αvβ3 integrin	Primary BMMs	(Singh et al., 2018)
BSP	Osteoclast surface, number, migration and bone resorption (+)	RGD sequence interact with αvβ3 integrin	BSP^−/−^ mice BSP^−/−^ preosteoclast	(Boudiffa et al., 2010)
OPN and BSP	Osteoclast number and bone resorption (+)	RGD sequence interact with αvβ3 integrin	OPN BSP DKO mice	(Bouleftour et al., 2019)

DKO, double knockout.

## Application of the ECM for Bone Tissue Engineering

Tissue engineering utilizes the basic principles and methods of life sciences and engineering to create functional tissue substitutes *in vitro*, which can be used to repair tissue defects and replace the partial or total loss of organ function (Shafiee and Atala, 2017). Tissue-engineering strategies rely on three basic elements—seed cells, scaffolds, and cytokines—which interact to produce engineered tissue constructs (Hu, 1992). Most tissue engineering approaches rely on renewable seed cells, such as stem cells, to restore damaged sites. The production of large amounts of growth factors and ECM components during the proliferation of seed cells increases the flexibility of the scaffold and promotes the proliferation and differentiation of autologous progenitor cells, thereby further enhancing tissue repair. Furthermore, cytokines bind to receptors on the cell surface, which transmit extracellular signals to the cell interior to regulate cell proliferation and differentiation, or enhance the formation of the ECM (Zhang et al., 2016). The scaffold provides an appropriate three-dimensional (3D) structure that guides the growth of seed cells to achieve correct tissue remodeling. Ideal scaffolds must have good biocompatibility, biodegradability, biomechanical properties, permeability, surface characteristics, and must not promote immune rejection (Yi et al., 2017).

In recent years, bone tissue engineering has developed rapidly, providing a promising new approach for bone repair. However, due to the complex anatomical structure of bone and the high mechanical stress that the engineered tissue must withstand *in vivo*, bone tissue regeneration remains one of the major challenges of tissue engineering (Vieira et al., 2017). Bone grafts can be used to stimulate or increase the formation of new bone around fractures or surgical implants, as well as to regenerate or replace the bone lost due to infection, trauma, or disease (Polo-Corrales et al., 2014). The ideal scaffold should also promote the attachment, increase the viability and proliferation, as well as induce osteogenic differentiation and angiogenesis. Finally, the material must be able to gradually integrate with the host tissue and bear the same load (Roseti et al., 2017). Bone scaffolds are usually made of biodegradable materials that are porous and effectively integrate seed cells, growth factors, and drugs, as well as provide mechanical support during the repair and regeneration of the damaged bone (Bose et al., 2012).

**Figure 1:**
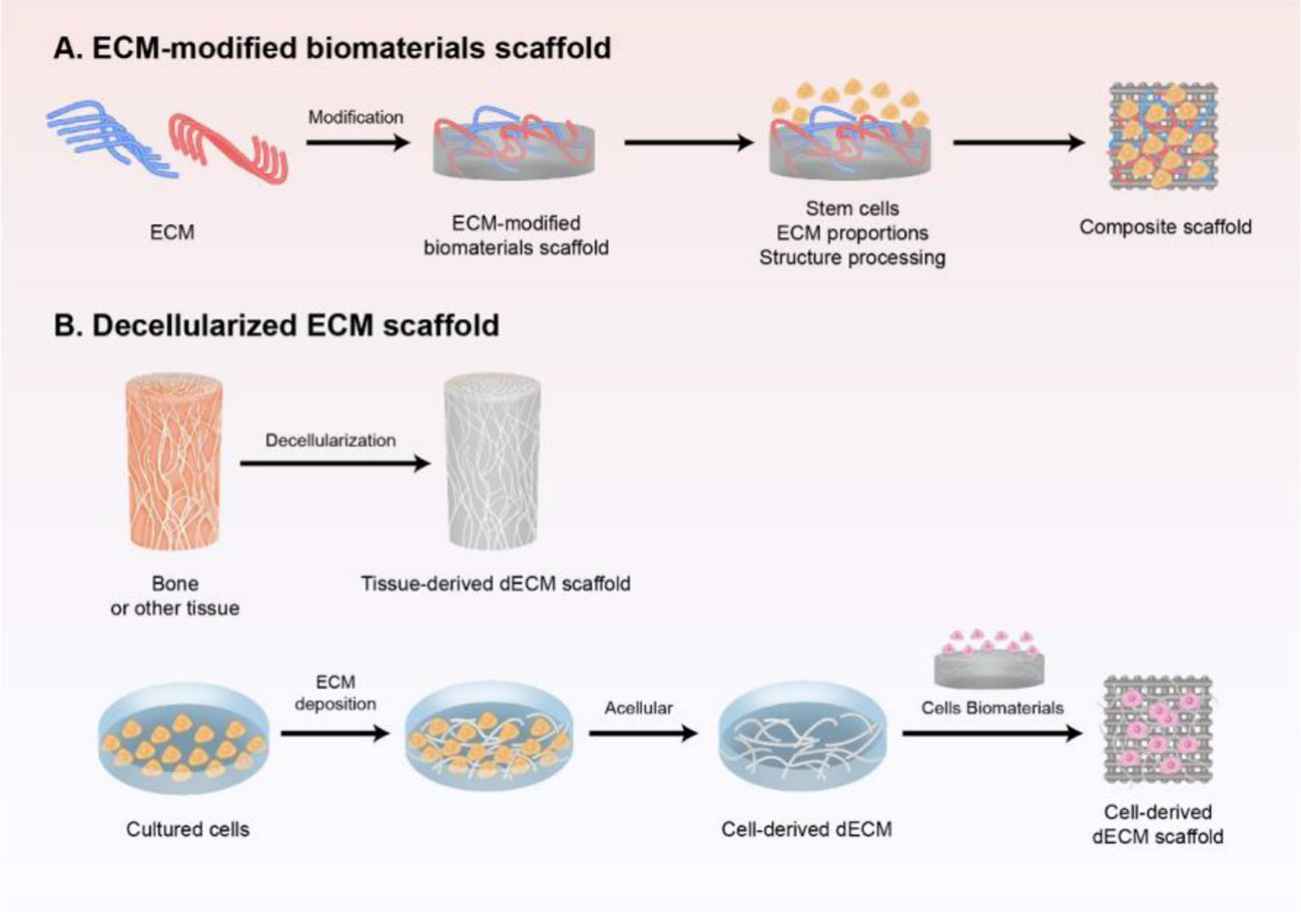
Schematic preparation of ECM-based scaffold in bone regeneration. **(A)** ECM-modified biomaterials scaffold. Different components and contents of ECM modified with biomaterial-based scaffold, and further modified with stem cells and structure processing to mimic the natural biomaterials. **(B)** decellularized ECM scaffold obtained either from tissue *in vivo* or cultured cells *in vitro* by decellularization, which is a promising strategy to induce bone regeneration and has good clinical performance.

With the rapid development of regenerative medicine, the ECM has gained attention as the fourth element in the development of bone tissue engineering (Ravindran et al., 2012) (**Figure 1**). The ECM acts as a physical scaffold and substrate for cell adhesion, delivering biochemical and biomechanical signals for cells to initiate migration, differentiation, morphogenesis, and homeostasis (Yi et al., 2017).

### ECM-Modified Biomaterial Scaffold

Extracellular matrix components such as collagen, HA, and fibronectin are commonly used as natural biomaterials for the preparation of scaffolds. ECM itself or modified with biomaterial-based scaffold is used in biological scaffolds to mimic the natural biomaterials. Because a single bone ECM component cannot generally simulate the complex osteogenic microenvironment, two or more materials are used to generate a composite that can produce a synergistic effect.

ECM act as a surface coating material on absorbable polymers and is increasingly being used to manufacture biodegradable scaffolds for bone reconstruction materials. Rentsch et al. constructed polycaprolactone-co-lactide (PCL) scaffolds coated with 3D collagen I/chondroitin sulfate (Coll I/CS) to repair rabbit calvarial bone defects. Compared with PCL scaffolds, more new bone was formed in the central defect of the Coll/CS coated PCL group, and it was more evenly distributed in the scaffolds after 6 months following implantation (Rentsch et al., 2014). In addition, titanium (Ti) was coated with Col1 and implanted into the femoral condyles of osteopenic rats to evaluate the osteointegration, the total bone ingrowth of the TiColl material following ovariectomy increased significantly from 4 to 12 weeks after implantation, compared with Ti alone (Sartori et al., 2015). Interestingly, the osteogenic potential of hydroxyapatite/β‐tricalcium phosphate (HA/β‐TCP) was improved by surface immobilization of MEPE peptide. The HA/β‐TCP with the MEPE peptide stimulated bone regeneration in a mouse calvarial defect model compared to unmodified HA/β‐TCP. Newly formed bones undergo physiological remodeling mediated by osteoclasts (Acharya et al., 2012). Therefore, due to the special structure and function of ECM, it might be beneficial for the biopolymer scaffold to perform signal connection and conduction with cells, improve the osteoconduction and osteointegration, and guide cell growth and tissue remodeling.

As an important ECM component of natural bone tissue, HA has also been used in materials for bone regeneration and bone repairs, such as bone fillings and injectable bone substitutes. A HA modified PCL/HA composite had better biocompatibility for hMSCs cells with higher proliferation and osteogenic potential, compared to neat PCL. Whereby the efficiency of attachment between hMSCs and the PCL/HA scaffold was improved with a higher HA content of 5% to 10% and in a HA concentration-dependent manner (Kumar et al., 2017). This means that in addition to the different components of modified ECM to affect the cell behaviors in bone regeneration, different ECM contents also play different roles.

In bone tissue engineering, biological scaffolds are required not only to have components similar to natural bone, but also to have similar structural properties. A collagen-apatite (Col-Ap) nanocomposite that emulates bone-like subfibrillar nanostructures was constructed to mimic natural bone. The Col-Ap nanocomposite scaffold was able to activate bone-forming cells, promote inward vascularization, as well as induce the synthesis of the ECM mediated by increased TGFβ1. (Liu et al., 2016). In addition, Haj et al. demonstrated that nanofibrous HA/chitosan (nHAp/CTS) scaffolds seeded with MSCs were superior to membranous HAp/CTS in a rat model of cranial bone defect regeneration. The MSCs in the nanofibrous scaffold activated the integrin-BMP/Smad signaling, leading to higher proliferation and ALP activity (Liu et al., 2013). Similar to nanofibrous HA scaffold, Shamaz et al. obtained electrospun microfibrous sheets by combining layers of a microfibrous mat composed of electrospun poly(l-lactic acid) (PLLA), gelatin–nanoHA matrix (GHA), and 1-ethyl-3-(3-dimethylaminopropyl) carbodiimide called GHA-MF_E_. When human adipose-derived MSCs (hADMSCs) were grown on this GHA-MF_E_ scaffold, they displayed higher ALP activity *in vitro*. Moreover, the GHA-MF_E_ fiber scaffolds significantly increased the rate of new bone formation in rabbit femoral cortical bone defect after 4 weeks of implantation compared with commercial Surgiwear™ (Shamaz et al., 2015). Obviously, the surface morphology and overall topology of ECM in scaffolds are significantly involved in determining their capacity for cell loading and growth in bone tissue engineering.

Stem cells are receiving increasing attention in regenerative medicine, including bone regeneration. Because of their good proliferation ability and capacity for osteogenic differentiation. On the other hand, stem cells are capable of synthesizing an ECM that can accelerate calcification and repair, thereby restoring the function of damaged bones (Clough et al., 2015; Gao et al., 2017). Chamieh et al. treated critical-size calvarial defects in rats using human dental pulp stem cells (DPSCs) seeded onto collagen gel scaﬀolds. Compared to untreated defects, the scaffolds containing DPSCs significantly promoted the formation of correctly structured new bone and increased the volume of fibrous connective tissue and mineralized tissue, which was accompanied by the increased expression of osteogenic ALP and type I collagen (Chamieh et al., 2016). When MSCs on laminated HA nanoparticle (nHA)/poly-hydroxybutyrate (PHB) (nHA/PHB) were co-implanted, it resulted in improved promoted the formation of osteoid tissue and ECM, with ingrowth of blood vessels into the graft two months after subcutaneous implantation on the dorsal site of mice model (Chen et al., 2017). Moreover, MSCs derived from induced pluripotent stem cells (iPSC-MSCs) combined with HAp/Col/CTS nanofibers also had a good bone regeneration ability in mice cranial defects, with almost 2-fold higher bone density than either TCP, CTS or HAp/CTS scaffolds. This might due to increased secretion of Alp and Col (Xie et al., 2016). On account of the synergistic effect of stem cells and ECM, the stem cells/ECM composite scaffolds are more conducive to bone remodeling than ECM modified scaffolds. Besides stem cells, endothelial cells (ECs) that contribute to vascularization can provide adequate nutritional support for the scaffold. Osteogenic differentiated MSCs (OMSCs) and ECs were seeded into a nano‐HA/polyurethane (n‐HA/PU) scaffold at a ratio of 0.5/1.5, was more effective for bone repair in rat condylar femoral defects than OMSC scaffold and scaffold alone. Therefore, ECs in OMSC/EC‐scaffold plays an important role in bone formation and vascularization (Li et al., 2019).

In the clinical study, the absorbable collagen sponge scaffold contains bone-stimulating agents, such as rhBMP-2, rhBMP-7, and PRP, to treat long bone defects and fracture of the patient. The patients showed bony healing and new bone formation in the defect site (Govender et al., 2002; Calori et al., 2008). Except for collagen, controlled proportions of HA together with modified calcium phosphate, TCP, and ionic species to form Bonelike^®^, which can be used in non-critical bone defects treatment. Bonelike^®^ has a similar chemical and structural composition of human bone. Bonelike^®^ itself or combined with MSCs improved bone regeneration by promoting bone growth and vascularization in bone defect patients (Campos et al., 2019). Moreover, eggshell-derived nano-hydroxyapatite for bone transplantation has strong safety and can obtain good bone regeneration performance. In the third month after implantation in patients, bone graft showed increased bone density and complete healing (Kattimani et al., 2019). Therefore, the use of ECM-modified scaffold in bone regeneration is significantly better than standard treatment by reducing the frequency of secondary intervention, while reducing the infection rate in patients with an open bone defect.

Above all, different types, proportions, structures of ECM, and even different implanted cells can all affect the bone regeneration performance of the ECM-modified biomaterial scaffold, suggesting that there may be a set of elements of ECM that work in concert to guide bone regeneration. Moreover, it remains unknown how much each of these factors or the combination of these factors contributes to ECM in the scaffold. Further studies are still needed to fully reveal the multiple functions of ECM in the ECM-modified biomaterial scaffold during bone repair.

### Decellularized ECM Scaffold

Although the ECM-modified biomaterial scaffold based on different compositions and ratios of bone ECM can improve bone defect repair, the complex matrix components and activities cannot be completely stimulated in biomimetic bone tissue. In addition, these artificial scaffolds lack specific cell niche and anatomical structures of target tissues, and cannot guarantee good integration of cellular and molecular cues (Zhang et al., 2016). Therefore, decellularized ECM scaffold obtained either from tissue *in vivo* or cultured cells *in vitro* is a promising strategy to induce bone regeneration and has a good clinical performance. It has the advantage of maintaining ECM components, providing the original geometry and flexibility of the tissue, while also offering inherently low immunogenicity (Hoshiba et al., 2016). The decellularized ECM provides mechanical support for the regenerating cells and affects both their migration and cell fate decision (Gallie et al., 1989).

#### Tissue-Derived Decellularized ECM Scaffold

Bone-derived decellularized ECM (dECM) can provide a native microenvironment containing ECM proteins, type I collagen, and growth factors including bone morphogenetic proteins. Kim et al. used dECM from porcine bone to form 3D-printed PCL/β-TCP/bone dECM scaffolds, which promoted more new bone regeneration 6 weeks after repair of a rabbit calvarial defect *in vivo*. Importantly, bone tissue developed into the interior of the scaffold. By contrast, bone tissue formed only at the edge of the scaffold without dECM (Kim et al., 2018). A dECM derived from the porous growth plate (GP) was fabricated to repair critical-sized rat cranial defects. Higher levels of mineralized tissue and increased vascular volume were observed 8 weeks after implantation, which might be caused by reduced production of IL-1β and IL-8 and superior osteogenic capacity compared to native GP (Cunniffe et al., 2017). In addition, 3D ECM scaffold produced from decellularized periosteum promoted bone mineralization by controlling the size and direction of mineral crystals in rabbit bone defect regeneration, suggesting the crucial role of periosteum ECM in efficient healing of fractures and bone regeneration (Lin et al., 2018). In clinical, decellularized bone ECM from bovine trabecular bone discs with patient autogenous MSCs could treat distal tibia fracture. After 6 months, active bone formation can be detected in both callus and graft of the patient (Hesse et al., 2010). This means that native decellularized bone transplantation has a broad application prospect in orthopedic surgery.

A dECM produced from non-bone tissue can also be used in bone regeneration. Mohiuddin et al. demonstrated that a combination of decellularized adipose tissue (DAT) with adipose-derived stromal/stem cells (ASCs) is effective in the regenerative bone repair of mice critical-size femur defects. The group treated with the DAT hydrogel showed a higher deposition of OPN and collagen I as well as a higher bone area than the untreated group (Mohiuddin et al., 2019). Beyond that, porcine small intestinal submucosa (SIS) ECM was combined with true bone ceramic (TBC) and mineralized, to fabricate the tissue-derived ECM scaffold mSIS/TBC. This scaffold promoted the viability, proliferation, and osteogenesis of rat MSCs through the ERK1/2 and Smad1/5/8 signal pathways *in vitro*. Most importantly, bone formation in a rat critical size cranial defect model was greatly improved by the mSIS/TBC scaffold compared to a pure TBC scaffold (Sun et al., 2018). Taken together, the abundance of multiple ECM components in dECM from the tissue is an ideal biomaterial for bone tissue engineering.

#### Cell-Derived Decellularized ECM Scaffold

Autologous cells grown aseptically *in vitro* can be used to produce a cell-derived decellularized ECM avoiding the disadvantages of a tissue-derived decellularized ECM. ECM scaffolds derived from stem cells and bone cells can potentially better mimic the native bone microenvironment, thereby inducing bone regeneration (Sun et al., 2018). In vitro, adipose‐derived stem cells (ASCs) on hMSCs derived decellularized ECM showed more osteogenic colonies, accompanied by increased expression of osteogenic markers (Zhang et al., 2015). dECM derived from co-cultured MSCs and HUVECs promoted the osteogenic and angiogenic potential of BMSCs. Moreover, the 1/3 ratio of MSCs/HUVECs has the best angiogenic effect on MSCs (Carvalho et al., 2019b). Cell-derived dECM, rich in collagen, matrix macromolecules, and growth factors, has good biocompatibility and biodegradability, making it beneficial for the proliferation and osteogenic differentiation of MSCs, and can be used as cell culture matrix for bone regeneration medicine.

In bone repair applications, cell-derived dECM combined with inorganic material to composite hybrid scaffolds, providing stronger osteoinductive properties and mechanical support. The implantation of osteogenic ECM sheets (OECMS) that retain the native collagen I and growth factors, together with HA, enhanced bone regeneration in a rat model of femoral non-union at 5 and 8 weeks. The OECMS contained TGF-β and BMP2, leading to increased osteoinduction and osteoconduction (Onishi et al., 2018). When a dECM derived from MG63 cells was deposited on a CS/PCL scaffold, hMSCs exhibited enhanced attachment, proliferation, and osteogenic differentiation, and the scaffold showed anti-inflammatory features *in vitro*. Moreover, the dECM-coated CS/PCL demonstrated a good bone regeneration ability after *in vivo* implantation in rat calvarial defects, which was associated with increased mineralized tissue (Wu et al., 2019). According to the characteristics of different biomaterials and the good osteoinduction of ECM, tissue-engineered grafts can be customized to overcome the limitations of autograft and allograft.

Beyond that, dECM scaffolds for bone repair can also be obtained from other, non-bone cells. A PLGA/PLA scaffold was coated with dECM form human lung fibroblasts (hFDM) in bone defect repair by delivering BMP-2. The dECM/PLGA/PLA scaffold significantly promoted new bone formation in a rat model of a calvarial bone defect. Notably, the addition of BMP-2 led to almost complete healing of bone defects (Kim et al., 2015). Mesenchymal stromal cells derived from human nasal inferior turbinate tissue (hTMSCs) were combined with a 3D-printed PCL/poly(lactic-co-glycolic acid) (PLGA)/β-TCP scaffold to form a mineralized ECM scaffold. The corresponding implants improved bone formation in ectopic and orthotopic rat models compared to the bare scaffold, in accord with the increased osteogenic differentiation of hTMSCs on 3D-printed hybrid scaffolds *in vitro* (Pati et al., 2015). Further development of 3D printing technology in ECM-based scaffolds is beneficial to the field of bone tissue engineering and regenerative medicine.

## Conclusions and Prospects

Although natural bone grafts from autologous or allogeneic sources are the best choice for bone defect repair, their clinical applications are limited due to complications during surgery related to their sourcing. With the development of tissue engineering technology, biomaterials manufactured using materials engineering, nanotechnology, and 3D printing been used to develop novel implants for bone regeneration. However, many such novel materials suffer from shortcomings such as poor biocompatibility, low osteoinductivity, and high immunogenicity. ECM scaffolds have unique advantages in all these areas. Because they can better simulate the composition, distribution, and biochemical signals of various matrix components in native bone tissue, they can emulate the natural bone microenvironment. Consequently, such materials can effectively support bone regeneration and guide tissue reconstruction. Common ECM-modified scaffold designs use a single or a combination of components of the ECM or apply a coating combined with biomaterials to produce scaffolds. Even when using decellularized preparations of autologous or allogeneic tissue or cells cultured *in vitro*, the integrity and mechanical properties of the matrix components are preserved, while achieving low immunogenicity by removing cell-bound antigens. Bone ECM has been demonstrated to enhance bone regeneration. Therefore, the application of the ECM-modified biomaterial scaffold and decellularized ECM scaffold has become a new frontier in tissue engineering and regenerative medicine.

Nevertheless, the clinical application of ECM-modified biomaterial scaffold or decellularized ECM scaffold in bone repair still faces many problems, such as the preservation of growth factors and biochemical signals in the ECM during decellularization, modification of the ECM, design, and processing of ECM scaffolds, and standardization and mass production for clinical studies. There are decellularization methods that retain the characteristics and functions of the ECM. However, due to the complexity and dynamics of its components, there has been no systematic analysis of the components of the ECM secreted by cells or tissues, and it is not clear if decellularized ECM can completely match the biochemical imprint of the native bone ECM. Therefore, the components and composition of decellularized ECM scaffolds, as well as the dynamic changes of ECM under different culture conditions should be further studied to make it more similar to the natural ECM composition. Additionally, it is difficult to precisely control the ECM components secreted by cells, so that they can be standardized and unified in mass production. Cells can be genetically modified to express specific products in a timely and quantitative manner, and appropriate bioreactors can be used to monitor cell growth and product secretion. Consequently, ECM release standards can be established to improve the quality of the graft. Finally, the ECM can be modified by adding growth factors and bioactive molecules during the preparation of ECM scaffolds to improve the effectiveness of bone defect repair. Therefore, the types and amounts of bioactive molecules need to be further studied. While additives can enhance the bone regeneration ability of the defect site, they must not affect the growth of other adjacent tissues at the graft site, hence avoiding inflammation and hyperplasia. In addition, ECM scaffolds can be combined with autologous pluripotent stem cells or organ-specific progenitor cells for a better therapeutic effect. Finally, the design and processing of ECM scaffolds can make them fill the defect site more accurately, offering better mechanical support and functional bionics. With the development of 3D printing technology in recent years, the ECM can be processed through biological printing to obtain scaffolds with various topology, such as porous and lamellar, or even scaffolds with a shape that exactly matches the defect site. Thus, the implant can be designed for improved bionic mechanical properties and stronger bone regeneration ability.

In conclusion, the application of ECM in bone formation and bone regeneration is full of opportunities and challenges. In the future, further studies on the cellular and molecular mechanisms the mediate the effects of the ECM on bone cells and bone repair will contribute to the further development of ECM-based scaffolds in bone tissue engineering.

## Author Contributions

XL and SP drafted the manuscript. AQ and Y-GG designed the project.

## Conflict of Interest

The authors declare that the research was conducted in the absence of any commercial or financial relationships that could be construed as a potential conflict of interest.
